# Noise suppression in photon-counting computed tomography using unsupervised Poisson flow generative models

**DOI:** 10.1186/s42492-024-00175-6

**Published:** 2024-09-23

**Authors:** Dennis Hein, Staffan Holmin, Timothy Szczykutowicz, Jonathan S. Maltz, Mats Danielsson, Ge Wang, Mats Persson

**Affiliations:** 1 Department of Physics, KTH Royal Institute of Technology, Stockholm, 1142 Sweden; 2 MedTechLabs, Karolinska University Hospital, Stockholm, 17164 Sweden; 3 Department of Clinical Neuroscience, Karolinska Institutet, Stockholm, 17164 Sweden; 4 Department of Neuroradiology, Karolinska University Hospital, Stockholm, 17164 Sweden; 5 Department of Radiology, School of Medicine and Public Health, University of Wisconsin, Madison, WI 53705 United States; 6 GE HealthCare, Waukesha, WI 53188 United States; 7 Department of Biomedical Engineering, School of Engineering, Biomedical Imaging Center, Center for Biotechnology and Interdisciplinary Studies, Rensselaer Polytechnic Institute, Troy, NY 12180 United States

**Keywords:** Deep learning, Photon-counting CT, Denoising, Diffusion models, Poisson flow generative models

## Abstract

Deep learning (DL) has proven to be important for computed tomography (CT) image denoising. However, such models are usually trained under supervision, requiring paired data that may be difficult to obtain in practice. Diffusion models offer unsupervised means of solving a wide range of inverse problems via posterior sampling. In particular, using the estimated unconditional score function of the prior distribution, obtained via unsupervised learning, one can sample from the desired posterior via hijacking and regularization. However, due to the iterative solvers used, the number of function evaluations (NFE) required may be orders of magnitudes larger than for single-step samplers. In this paper, we present a novel image denoising technique for photon-counting CT by extending the unsupervised approach to inverse problem solving to the case of Poisson flow generative models (PFGM)++. By hijacking and regularizing the sampling process we obtain a single-step sampler, that is NFE = 1. Our proposed method incorporates posterior sampling using diffusion models as a special case. We demonstrate that the added robustness afforded by the PFGM++ framework yields significant performance gains. Our results indicate competitive performance compared to popular supervised, including state-of-the-art diffusion-style models with NFE = 1 (consistency models), unsupervised, and non-DL-based image denoising techniques, on clinical low-dose CT data and clinical images from a prototype photon-counting CT system developed by GE HealthCare.

## Introduction

X-ray computed tomography (CT) is a medical imaging modality used for the diagnosis and treatment planning of a wide range of diseases, including stroke, cancer, and cardiovascular diseases. However, because of the potential risks posed by even low doses of ionizing radiation, considerable efforts have been made to enable high diagnostic quality while maintaining a dose as low as reasonably achievable [1, 2]. Photon-counting CT (PCCT), which is based on the latest generation of CT detector technology, can reduce the dose via photon energy weighting and eliminate the effects of electronic noise. PCCT can also enable imaging with a higher spatial resolution and produce single-exposure energy-resolved images [3–6]. However, obtaining high resolution in either space or energy decreases the number of photons in each voxel or energy bin, which increases the image noise. Thus, an excellent noise performance is required, possibly exceeding the capabilities of today’s state-of-the-art denoising methods.

In recent years, deep learning (DL) methods have demonstrated remarkable success in low-dose CT (LDCT) and PCCT image denoising [7–19]. However, these methods are commonly trained using supervised learning, which requires paired data, which are not always available in practice. In particular, paired and perfectly registered clinical images are difficult to obtain, and methods for generating paired data based on simulating low-dose scanning [20] or adding noise maps from phantom scans [21] may be sensitive to imperfect modeling of the systems or mismatches between patient and phantom geometries. In PCCT, pulse pileup affects high- and low-dose scans differently, further confounding the training schemes. Therefore, unsupervised and self-supervised methods are becoming increasingly common [12, 13, 15–17].

Diffusion-style models, such as diffusion and Poisson flow models, have demonstrated considerable success in unconditional [22–29] and conditional image generation [25, 30–33]. Notably, for medical imaging, this family of models lends themselves very well to inverse problem solving via posterior sampling and has already been demonstrated on a range of inverse problems [17–19, 34, 35]. In particular, in the case of diffusion models, it is possible to manipulate the sampling process and retask the network, trained in an unsupervised manner, for inverse problem-solving [17, 31, 34, 35]. This is typically performed in two steps. First, information from the prior distribution, that is, the ground truth data, is obtained by estimating its time-dependent score function via denoising score matching, exactly as when training an unconditional generator. Once equipped with the estimated score function, samples can be drawn from the desired posterior distribution by augmenting the sampling process with a data consistency step that regularizes the generative process and forces the sample to be consistent with the input (conditioning) image. Moreover, running the sampling process using the initial sample from a prior noise distribution is unnecessary. Indeed, it is even beneficial to ‘hijack’ the sampling process by inserting a version of the condition image at some stage of the reverse diffusion [31, 35]. Hijacking will not only help regularize the problem further but will also result in faster sampling; a smaller number of function evaluations (NFE) are required to achieve the desired image quality. Note that this is an efficient strategy because the learned score function can be successfully used to solve a range of different inverse problems or downstream tasks without retraining [34, 35]. It is also possible to solve the inverse problem directly using supervised learning [18, 19]. However, the supervised approach requires paired data.

The NFE required for diffusion-style models, such as diffusion and Poisson flow models, may be of the order 10^1^–10^3^ for both conditional (image-to-image) and unconditional (noise-to-image) generation. This limits their use in applications where speed is critical, such as clinical CT image denoising. Efforts to reduce the required NFE include the use of efficient ordinary differential equation (ODE) samplers [26] and distillation techniques [36]. Consistency models [37] build on the probability flow ODE formulation of diffusion models and learn a consistency network that maps any point on the trajectory to its initial point, including the final point, which is a sample from the prior noise distribution. Thus, it achieves single-step sampling (NFE = 1). A consistency model may be distilled from a pre-trained diffusion model using so-called consistency distillation (CD) or obtained as a stand-alone model using consistency training.

This study presents a novel image denoising technique for low-dose and photon-counting CT that extends posterior sampling Poisson flow generative models (PPFM) [19] to cases where paired data are not available. The PPFM is a diffusion-style posterior sampling image denoising method that exploits the added robustness of the PFGM++ framework to enable NFE = 1 while maintaining high image quality. In particular, an unconditional PFGM++ was first trained for image generation on randomly extracted patches from the prior, ground truth, and images. The sampling process was subsequently hijacked and regularized, as in PPFM, to ensure consistency with the input, condition, and image. The main contributions of this study are as follows. (1) Unsupervised PPFM, a novel image-denoising technique that extends PPFM [19] to the case when paired data are unavailable, is presented. (2) It is demonstrated that it is possible to efficiently train the network in an unsupervised manner on patches extracted from ground truth data and subsequently manipulate the sampling process to denoise full-resolution images. Training on randomly extracted patches is more efficient in terms of graphics memory requirements and provides additional regularization. (3) The proposed method contains a posterior sampling diffusion model (EDM [27]) as a special case when *D* → ∞. The results indicate that the added flexibility of choosing D as a hyperparameter results in improved performance when *D* → ∞ as is the case for diffusion models. (4) The proposed method was evaluated using clinical low-dose CT (LDCT) images and clinical images from a prototype PCCT system developed by GE Healthcare (Waukesha, WI, USA) [38]. It is demonstrated that the proposed method performs competitively compared with current state-of-the-art diffusion-style models with NFE = 1 and consistency models [37]. Notably, the consistency model was trained in a supervised manner, whereas the proposed method was unsupervised. Despite imposing a significantly laxer data requirement, the proposed method performs both quantitatively and qualitatively. In addition to consistency models, the proposed method was compared with popular supervised, unsupervised, and non-DL-based image denoising techniques.

The code used in this study is available at: https://github.com/dennishein/pfgmpp_PCCT_denoising.

## Methods

### Diffusion models and PFGM++ 

Diffusion models (EDM [27]) and PFGM++ [29] work by iteratively denoising images by following a physically meaningful trajectory inspired by nonequilibrium thermodynamics and electrostatics, respectively. Despite the widely different underlying physics, diffusion models and PFGM++ are intimately connected in theory and practice. The training and sampling processes of PFGM++ converge to those of the diffusion models in the *D* → ∞, $r=\sigma \sqrt{D}$ limit [29]. Thus, diffusion models were incorporated as special cases of PFGM++. In addition, it is possible to reuse the training and sampling algorithms in the EDM [27] for PFGM++ using a simple change in variables and an updated prior noise distribution [29]. Expanding on the probability flow ODE formulation in ref. [25], the EDM [25] writes the ODE as

1 $$dx = -\dot{\sigma }(t)\sigma (t){\nabla }_{x}log{p}_{\sigma (t)}({\varvec{x}})dt$$

where ${\nabla }_{x}log{p}_{\sigma (t)}({\varvec{x}})$ is the time-dependent score function of the perturbed distribution and *σ*(*t*) is the noise scale. This ODE defines the trajectory between an easy-to-sample prior noise distribution, a simple Gaussian distribution, and the data distribution of interest. Intuitively, running the ODE forward or backward in time nudges the sample toward or away from the prior noise distribution. Notably, Eq. 1 depends only on the data distribution via the gradient of the log-likelihood, also known as the score function. Let *p*(***y***) represents the data distribution, *p*(*σ*) represents the distribution of noise scales, and ${p}_{\sigma }({\varvec{x}}|{\varvec{y}})=\mathcal{N}\left({\varvec{y}}, {\sigma }^{2}{\varvec{I}}\right)$ the Gaussian perturbation kernel. The score function can then be estimated using a perturbation-based objective:

$${\mathbb{E}}_{\sigma \sim p\left(\sigma \right)}{\mathbb{E}}_{{\varvec{y}}\sim p\left({\varvec{y}}\right)}{\mathbb{E}}_{{\varvec{x}}\sim {p}_{\sigma }\left({\varvec{x}}|{\varvec{y}}\right)}\left[\lambda \left(\sigma \right)\Vert {f}_{\theta }\left({\varvec{x}}, \sigma \right)-{\nabla }_{{\varvec{x}}}\text{log}{p}_{\sigma }{\left({\varvec{x}}|{\varvec{y}}\right)\Vert }_{2}^{2}\right]$$

where λ(σ) denotes a weighting function. Given the estimation of the time-dependent score function, a sample was generated by solving Eq. 1, using an iterative ODE solver, starting from an initial sample from the prior noise distribution.

PFGM++ [29] operates by treating N-dimensional data as electric charges in an (N + D)-dimensional augmented space. Let $\widetilde{{\varvec{y}}}:=\left({\varvec{y}}, 0\right)\in {\mathbb{R}}^{N+D}$ and $\widetilde{{\varvec{x}}}:=\left({\varvec{x}},\boldsymbol{ }{\varvec{z}}\right)\in {\mathbb{R}}^{N+D}$ denote the augmented ground truth and perturbed data, respectively. The object of interest in high-dimensional electric fields is

2 $${\varvec{E}}\left(\widetilde{{\varvec{x}}}\right)=\frac{1}{{S}_{N + D-1}\left(1\right)}\int \frac{\widetilde{{\varvec{x}}}-\widetilde{{\varvec{y}}}}{{\Vert \widetilde{{\varvec{x}}}-\widetilde{{\varvec{y}}}\Vert }^{N + D}}p\left({\varvec{y}}\right)d{\varvec{y}}$$

where S_N+D−1_(1) is the surface area of the unit (N + D − 1)-sphere and *p*(***y***) is the ground truth and data distribution. However, owing to the rotational symmetry of the D-dimensional cylinder,${\sum }_{i=1}^{D}{z}_{i}^{2}={r}^{2},\forall r>0$, dimensionality reduction is possible [29]. In fact, it suffices to track the norm of the augmented variables $r=r\left(\widetilde{{\varvec{x}}}\right):={\Vert z\Vert }_{2}$. For notational brevity, $\widetilde{{\varvec{y}}}:=\left({\varvec{y}}, 0\right)\in {\mathbb{R}}^{N+1}$ and $\widetilde{{\varvec{x}}}:=\left({\varvec{x}},\boldsymbol{ }\mathbf{r}\right)\in {\mathbb{R}}^{N+1}$ are redefined.

The ODE of interest is:

3 $$d{\varvec{x}}={{\varvec{E}}\left(\widetilde{{\varvec{x}}}\right)}_{{\varvec{x}}}\cdot {E\left(\widetilde{{\varvec{x}}}\right)}_{r}^{-1}dr$$

where ${{\varvec{E}}\left(\widetilde{{\varvec{x}}}\right)}_{{\varvec{x}}}$, and ${{\varvec{E}}\left(\widetilde{{\varvec{x}}}\right)}_{r}$, a scalar, denote the ***x*** and *r* components of ${\varvec{E}}\left(\widetilde{{\varvec{x}}}\right)$, respectively. Equation 3 defines a surjection between the data on the *r* = 0 hyperplane and an easy-to-sample prior noise distribution on the *r* = *r*_max_ hyper-cylinder [29]. For the diffusion models, PFGM++ employs a perturbation-based objective. Let *p*(*r*) be the training distribution over *r* and ${p}_{r}({\varvec{x}}|{\varvec{y}})$ be the perturbation kernel. Then, the objective of interest is

4 $${\mathbb{E}}_{r\sim p\left(r\right)}{\mathbb{E}}_{{\varvec{y}}\sim p\left({\varvec{y}}\right)}{{\mathbb{E}}_{{\varvec{x}}\sim {p}_{r}\left({\varvec{x}}|{\varvec{y}}\right)}{\Vert {f}_{\theta }\left(\widetilde{{\varvec{x}}}\right)-\frac{{\varvec{x}}-{\varvec{y}}}{r/\sqrt{D}}\Vert }_{2}^{2}}$$

Now, if ${p}_{r}\left({\varvec{x}}|{\varvec{y}}\right)\propto 1/{\left({\Vert {\varvec{x}}-{\varvec{y}}\Vert }_{2}^{2}+{r}^{2}\right)}^{\frac{N+D}{2}}$ then it is possible to show that the minimizer of Eq. 4 is ${f}_{\theta }^{*}\left(\widetilde{{\varvec{x}}}\right)=\sqrt{D}{{\varvec{E}}\left(\widetilde{{\varvec{x}}}\right)}_{{\varvec{x}}}\cdot {E\left(\widetilde{{\varvec{x}}}\right)}_{r}^{-1}$. As for diffusion models, a sample can be generated by solving $dx/dr={{\varvec{E}}\left(\widetilde{{\varvec{x}}}\right)}_{{\varvec{x}}}/{E\left(\widetilde{{\varvec{x}}}\right)}_{r}={f}_{\theta }^{*}\left(\widetilde{{\varvec{x}}}\right)/\sqrt{D}$, starting from an initial sample from the prior noise distribution, ${p}_{{r}_{max}}$, using an iterative ODE solver.

### Problem formulation

The problem of obtaining a high quality reconstruction $\widetilde{{\varvec{y}}}\in {\mathbb{R}}^{N}$ of ${\varvec{y}}\in {\mathbb{R}}^{N}$ based on noisy observations $\varvec{c}=\mathcal{F}\left({\varvec{y}}\right)\in {\mathbb{R}}^{N}$ is treated as a statistical inverse problem, where $\mathcal{F}:{\mathbb{R}}^{N}\to {\mathbb{R}}^{N}$ is a ‘catch-all’ noise degradation operator, including factors such as quantum noise [7], and *N*: = *n* × *n*. Thus, it is assumed that the data follow a prior distribution $y\sim p\left(y\right)$ and the objective is to enable sampling from the posterior $p\left({\varvec{y}}|{\varvec{c}}\right)$. This approach to inverse problem-solving is referred to as posterior sampling. In this study, ***y*** is treated as “ground truth” despite the fact that it may contain noise and artifacts.

### Image denoising via posterior sampling

The proposed method has two main components: (1) A learned PFGM++ trained in an unsupervised manner for unconditional image generation. (2) A sampling scheme that regularizes the generative process and enforces consistency with the input, conditions, and images. By combining the information from the prior distribution, *p*(***y***), embedded in the learned empirical electric, the field can be sampled with a modified sampling scheme to ensure data consistency, and sampled from the desired posterior *p*(***y***|***c***), thus providing a solution to the inverse problem. This strategy has been successfully applied to diffusion models for a range of inverse problems [17, 31, 34, 35]. The key idea of this study was to extend this approach to the case of PFGM++. Direct extension to PFGM++ is feasible because of its intimate connection with diffusion models. The theoretical analysis is left for future work and empirical support is provided for obtaining a sample from the desired posterior. In particular, it is demonstrated that $\widehat{{\varvec{y}} }\approx {\varvec{y}}$. Similar to PFGM++ [29], the proposed sampling algorithm is reused from ref. [27] with an updated prior noise distribution. This is feasible because of the hyperparameter translation formula, $r = \sigma \sqrt{D}$, $\widetilde{{\varvec{x}}}:=\left({\varvec{x}}, r\right)$, the fact that $\sigma \left(t\right)=t$ in ref. [27], and by a change of variable $d{\varvec{x}}={f}_{\theta }^{*}\left(\widetilde{{\varvec{x}}}\right)/\sqrt{D}dr={f}_{\theta }^{*}\left(\widetilde{{\varvec{x}}}\right)dt$ since $dr=d\sigma \sqrt{D}=dt\sqrt{D}$. The proposed sampling algorithm is detailed in Algorithm 1 with updates to sampling vs [29] highlighted in blue. The first update is hijacking, and instead of starting from an initial sample for the prior noise distribution, the reverse process is hijacked at some $i = \tau \in {\mathbb{Z}}_{+}$, *τ* < *T* and simply inject the condition image ***x***_τ_ = ***c***. In addition to hijacking, data consistency, regularization, and steps are considered. It must be updated for various inverse problems. For image denoising, simply using an identity map is sufficient. Thus, ***x***_*i*+1_ is simply mixed with ***x***_τ_ = ***c***, where ***c*** is the condition image with weight *w* ∈ [0, 1]. Note that the proposed sampling algorithm is identical to that used in ref. [19] except that the network has now been trained using unsupervised learning and thus does not take the condition image **c** as an additional input.

**Figure:**
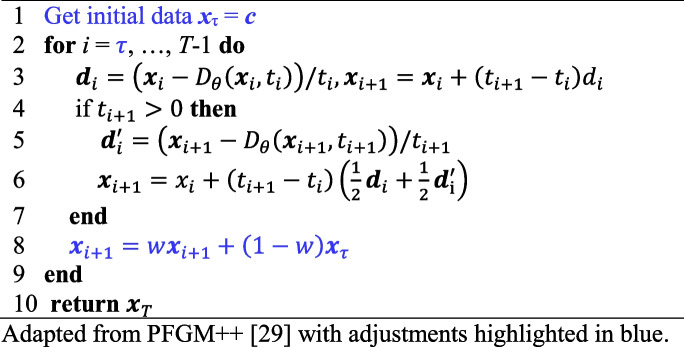
**Algorithm 1** Proposed PPFM sampling [29]

### Experiments

#### Datasets

Mayo LDCT data: For training and validation, a publicly available dataset from the Mayo Clinic used in the American Association of Physicists in Medicine (AAPM) Low-dose CT Grand Challenge [39] was used. This dataset contains images from ten patients reconstructed using medium (D30) and sharp (D45) kernels. Images were also available for 1 mm and 3 mm slice thicknesses. All the images had a matrix size of 512 × 512. In this study, a slice thickness of 1 mm and B30 reconstruction kernel were used. The first eight patients were used for training, yielding 4800 slices, and the last two for validation, yielding 1136 slices. Although paired normal-dose CT (NDCT) and LDCT images were available, only NDCT images were used for training the proposed method.

PCCT data: As test data, the images of two patients from a clinical study (Swedish Ethics Review Agency 2020–04638 and 2021–01092 and prospectively consented IRB review UW-IRB: 2022–1043) of a prototype silicon detector-based photon-counting system developed by GE Healthcare [38] were used. The patients were scanned at the Karolinska Institutet, Stockholm, Sweden (Case 1, effective diameter 28 cm, CTDI_vol_ = 10.12 mGy) and at the University of Wisconsin-Madison, Madison, WI (Case 2, effective diameter 36 cm, CTDI_vol_ = 27.64 mGy) using scan parameters listed in Table 1. Virtual monoenergetic images of 70 keV were reconstructed using filtered backprojection on a 512 × 512-pixel grid with a 0.42 mm slice thickness.

**Table 1:** Key parameters used for scanning patients on prototype PCCT systems

Parameter	PCCT (Case 1)	PCCT (Case 2)
Tube current	255 mA	290 mA
Helical pitch	0.990:1	0.510:1
Rotation time	0.6 s	0.7 s
kVp	120	120

#### Implementation details

Each network, *D* ∈ {64, 128, 2048} and *D* → ∞, was trained with a batch size of 32 using Adam [40] and a learning rate of 2 × 10^−4^ for 105 iterations. The DDPM++ architecture was used with a channel multiplier of 128 channels per resolution [1, 1, 2, 2, 2, 2, 2] and self-attention layers at resolutions of 16, 8, and 4. The preconditioning, exponential moving average schedule, and nonleaky augmentation suggested in ref. [27] with an augmentation probability of 15% were employed. Additionally, to prevent further overfitting, the dropout probability was set to 10%. To ensure efficient training, the network was trained on randomly extracted 256 × 256 patches. In other words, the unconditional generator was not trained to generate full-resolution CT images but rather patches extracted from CT images. In addition to facilitating efficient training, this served to further augment the dataset and, therefore, helped prevent overfitting. To reduce the graphics memory requirements, mixed precision was used in the training. Note that the same configuration, but with 512 × 512 images, would exceed the memory available on an NVIDIA A6000 48 GB GPU. To achieve NFE = 1, τ: = *T* − 1, was set. *T* and *w*, which are crucial hyperparameters in Algorithm 1, were set by grid search over *T* ∈ {4, 8, 16, 32, 64} and *w* ∈ {0.5, 0.6, 0.7, 0.8, 0.9, 1.0} using Learned Perceptual Image Patch Similarity (LPIPS) [41] on the validation was set as the selection criteria. The lowest (best) LPIPS was achieved for *T* = 8 and *w* = 0.5. Because *w* = 0.5 is a corner case, was also tried *w* = 0.4. However, this did not improve the performance. The proposed method is referred to as ‘single-step’ despite including a second step, regularization, since the time required from this operation is negligible and NFE = 1.

#### Comparison to other methods

The results were compared with those of popular non-DL-based, supervised, and unsupervised image-denoising techniques. For non-DL-based image denoising, a version of BM3D [42] was chosen because it has been shown to be the best performing method in the non-DL category for LDCT image denoising [7]. Bm3d.py (https://pypi.org/project/bm3d/) was used, and the parameter σ_BM3D_ was set by measuring the standard deviation in a flat region-of-interest (ROI) in the LDCT validation data. For the supervised techniques, the WGAN-VGG [9], consistency models [37], and PPFM [19] were used. The WGAN-VGG was trained on randomly extracted 64 × 64 from the LDCT training data with hyperparameters, as specified in ref. [9]. The consistency models [37] are state-of-the-art diffusion-style models with an NFE of 1. However, consistent models have been developed for generating unconditional images. Notably, ref. [19] was the first study to implement a consistency model for conditional (image-to-image) generation. It is reasonable to surmise that the ‘trick’ of feeding the condition image as additional input to the network to directly learn a trajectory to the posterior distribution of interest, a technique successfully used for diffusion models [30, 33, 43] and PFGM++ [19], will also work for CD. This hypothesis is empirically supported by ref. [19]. Minimal adjustments were made to the official implementation (https://github.com/openai/consistency_models) to feed conditional images as additional input. The network was trained on randomly extracted 256 × 256 patches from the LDCT data. Random rotations and mirrors were used for data augmentation. The hyperparameters for sampling and training were set as in the LSUN 256 × 256 experiments (https://github.com/openai/consistencymodels/blob/main/scripts/launch.sh), except for the batch size, which had to be reduced to four to fit in the memory of a single NVIDIA A6000 48 GB GPU. An EDM was first trained for 3 × 10^5^ iterations and then distilled into a consistency model for 6 × 10^5^ iterations. This model is referred to as CD. The training and sampling for the PPFM are specified in ref. [19]. The *D* = 64 case was used because this yields the best performance. The results for BM3D, WGAN-VGG, CD, and PPFM were derived directly from ref. [19]. Finally, as an example of an unsupervised method, Noise2Void [44] was used. The same hyperparameters as those for the BSD68 dataset in the original paper were used, using code from the official repository (https://github.com/juglab/n2v).

### Evaluation methods

For quantitative image quality assessment, the structural similarity index (SSIM) [45], peak signal-to-noise ratio (PSNR), and LPIPS [41] were employed. SSIM and PSNR are well-established metrics in medical imaging literature, but these relatively simple metrics do not necessarily correspond well to human perception [41]. For instance, the PSNR is inversely proportional to the *ℓ*_2_ Euclidean distance, and this simple pixelwise metric correlates well with human perception. This was particularly evident in the case of over-smoothing. To overcome some these issues, ref. [41] suggests using pretrained convolutional neural networks (CNNs) as feature extractors, as is the case for perceptual loss functions. The resulting metrics, called LPIPS, were shown to correspond more closely to human perception than more traditional metrics such as SSIM and PSNR in a range of experiments. In this study, the official implementation of LPIPS (https://github.com/richzhang/PerceptualSimilarity) with AlexNet [46] was used as the feature extractor.

**Fig. 1:**
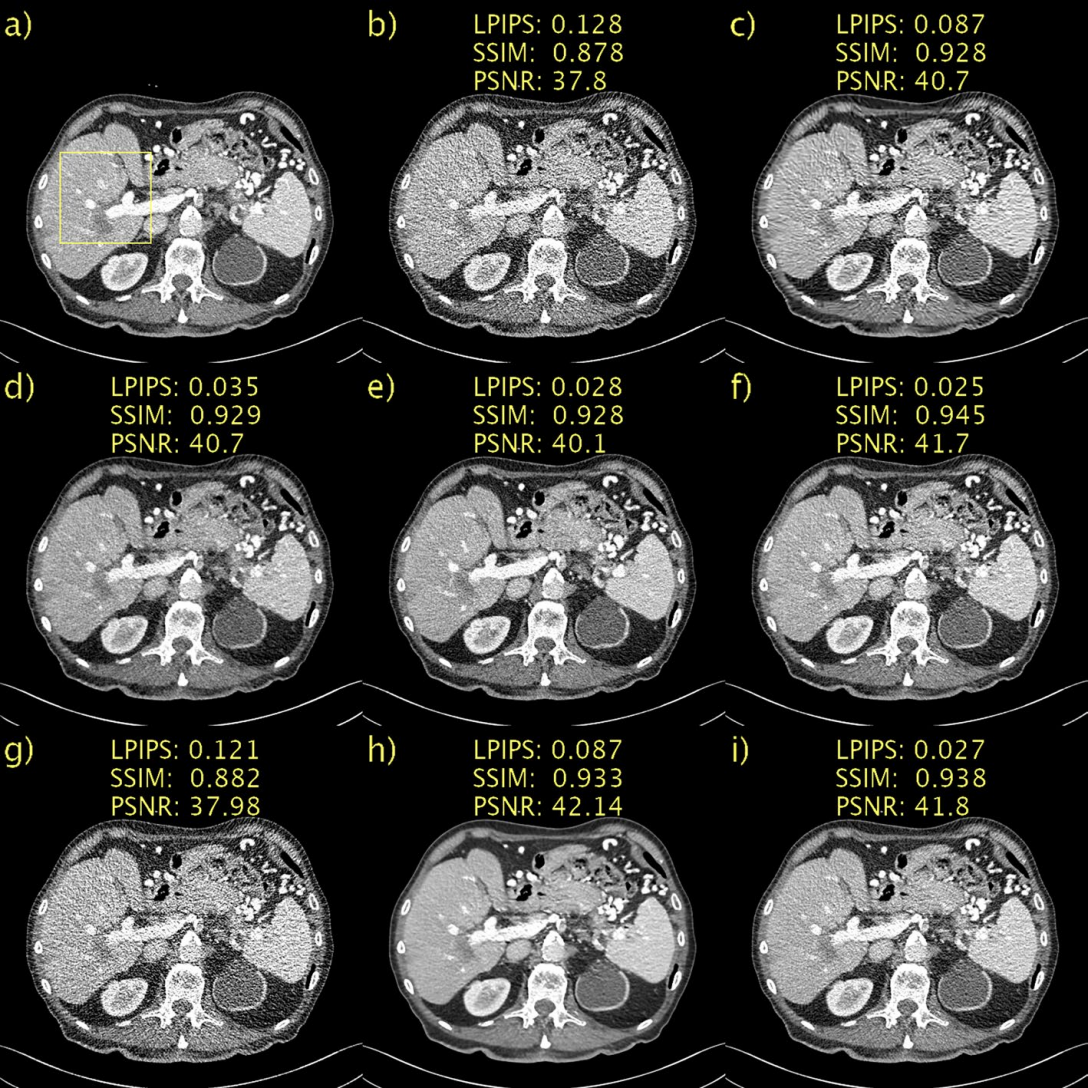
Results on the Mayo LDCT dataset. Abdomen image with a metastasis in the liver. **a** NDCT; **b** LDCT; **c** BM3D; **d** WGAN-VGG; **e** CD; **f** PPFM; **g** Noise2Void; **h** *D* → ∞; **i** *D* = 128. Yellow box indicating ROI shown in Fig. 2. 1 mm-slices. Window setting [-160, 240] HU

In addition to the evaluation with numerical metrics, two radiologists with 6 and 25 years of experience respectively performed a visual assessment of the image quality of the denoised images. CD was used as the reference, and the observers were asked to grade the image quality resulting from each of the six denoising methods relative to the CD image for the LDCT image in Fig. 1 and the PCCT images in Figs. 4 and 6 (in total 18 comparisons). Grading was performed on a five-point Likert scale ranging from -2 (significantly worse) to +2 (significantly better) in five categories: noise, contrast, sharpness, artifacts and overall image quality. The unprocessed PCCT image and the regular LDCT and NDCT images are also displayed for reference.

## Results

**Fig. 2:**
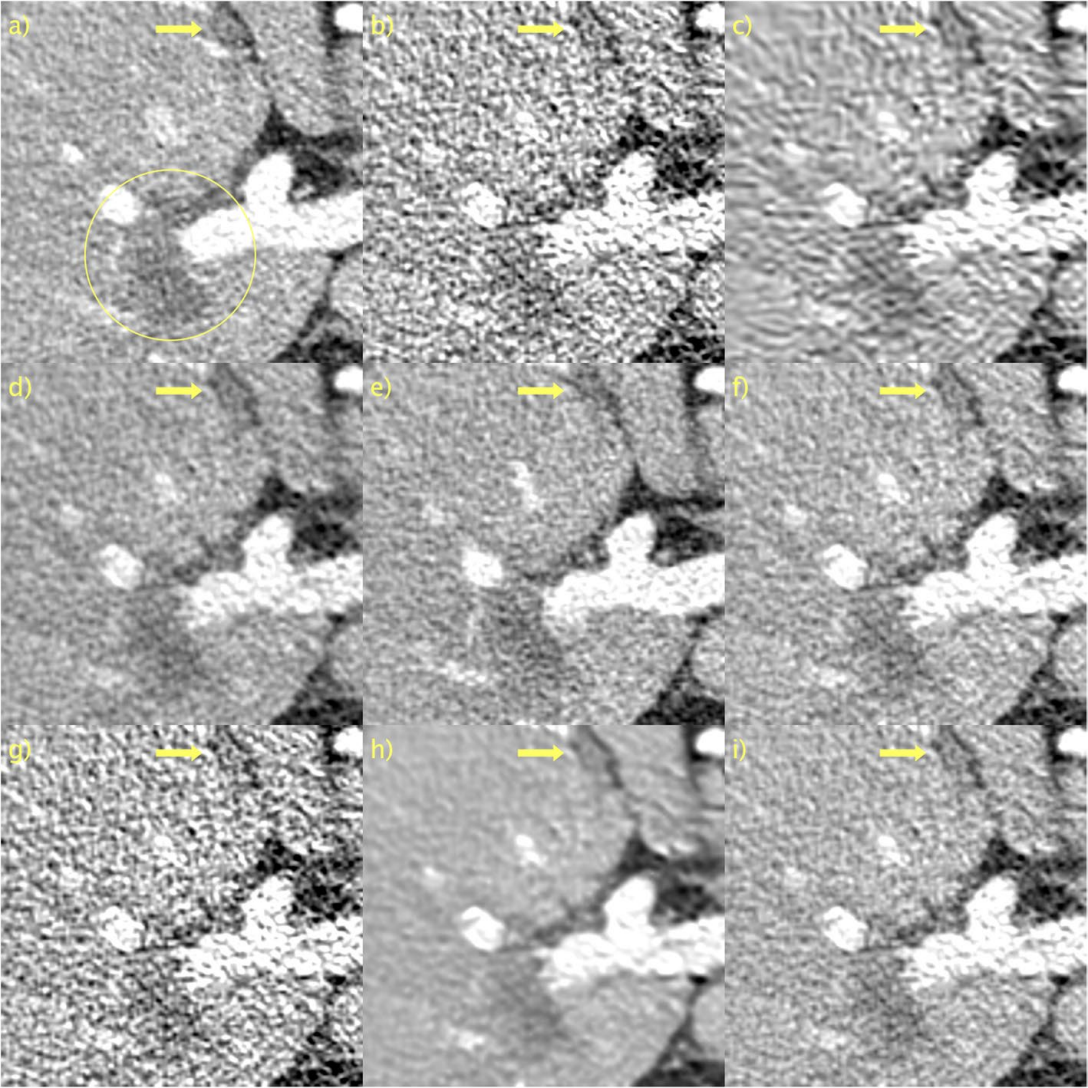
ROI in Fig. 1 magnified to emphasize details. **a** NDCT; **b** LDCT; **c** BM3D; **d** WGAN-VGG; **e** CD; **f** PPFM; **g** Noise2Void; **h** *D* → ∞; **i** *D* = 128. Yellow circle added to emphasize lesion. 1 mm-slices. Window setting [-160, 240] HU

The quantitative results, mean, and standard deviation of the LPIPS [41], SSIM [45], and PSNR for the LDCT validation set are listed in Table 2. Additionally included is the average time, in seconds, to process a single slice. The notebook used from the official implementation of Noise2Void does not utilize GPU acceleration, which should be considered when comparing the evaluation speeds. The overall top performer was PPFM. However, as mentioned above, SSIM and PSNR do not necessarily correspond closely to human perception. As expected, the proposed method is bounded in performance by PPFM because it is unsupervised. Moreover, it can be seen that the proposed method for *D* = 128 and *D* = 64 is better in terms of LPIPS than WGAN-VGG, a supervised method. Compared with CD, a state-of-the-art diffusion style model with NFE = 1, the performance of the proposed method was slightly worse. Importantly, CD was trained in a supervised manner, whereas the proposed method was unsupervised. Thus, the proposed method with *D* = 128 performed competitively. Comparing the two unsupervised methods, Noise2Void and the proposed method, it can be seen that the latter performs favorably. Noise2Void yields only a marginal improvement over LDCT images. The qualitative results, along with LPIPS, SSIM, and PSNR, for a representative slice from the Mayo LDCT validation data are shown in Fig. 1. For brevity, the results for the proposed method are shown only for *D* → ∞, and the best performance, *D* = 128. The former is included as an interesting case because it corresponds to a diffusion model instead of PFGM++. This patient had liver metastasis, and magnification of the ROI were included, as shown in Figs. 2a and b for NDCT and LDCT images, respectively, for reference. BM3D, as shown in Fig. 2c, performed well in terms of noise suppression while preserving the salient details. Nevertheless, this comes at the cost of artifacts that make the image appear smudgy. WGAN-VGG, CD, and PPFM, all supervised methods, as shown in Figs. 2d, e, and f, suppress noise effectively and keep the key details intact. A yellow arrow is overlaid to indicate the detail that appears in CD, shown in Fig. 2e, but in none of the other images, including NDCT and LDCT. It appears that CD has added a feature to the image that appears realistic, but that is not genuine, given that LDCT and NDCT images are used as references. Such inaccurate removal or addition of details is loosely referred to as a hallucination [2]. Noise2Void, shown in Fig. 2g, appears to essentially reproduce the LDCT image. However, quantitatively, there was only a marginal improvement. However, as shown in Fig. 2i, the proposed method (with *D* = 128) effectively suppressed noise while keeping the salient features intact. Qualitatively, it is difficult to discriminate between the proposed method with *D* = 128, which is an unsupervised method, and PPFM, which is shown in Fig. 2f, a supervised method. Quantitatively, for this particular slice, it can be seen that PPFM performs slightly better. Comparing *D* → ∞, shown in Fig. 2h, with *D* = 128 in Fig. 2i demonstrates the performance gains afforded by the PFGM++ framework. In particular, the proposed method with *D* → ∞ appeared over-smoothed and somewhat blurred.

**Table 2:** Mean and standard deviation of LPIPS, SSIM, and PSNR in the LDCT validation set, along with average time, in seconds, to evaluate a single slice

Method	LPIPS (↓)	SSIM (↑)	PSNR (↑)	Average time per slice (↓)
LDCT	0.075 ± 0.02	0.94 ± 0.02	41.5 ± 1.6	
BM3D [42]	0.050 ± 0.01	**0.97** ± 0.01	45.0 ± 1.6	3.38 × 10^0^
WGAN-VGG [9]	0.019 ± 0.01	0.96 ± 0.01	43.2 ± 0.9	1.34 × 10^–3^
CD [37]	0.013 ± 0.00	0.96 ± 0.01	43.1 ± 1.0	4.44 × 10^–2^
PPFM [19]	**0.010** ± 0.00	**0.97** ± 0.01	**45.4** ± 1.4	1.25 × 10^–2^
Noise2Void [44]	0.069 ± 0.02	0.94 ± 0.02	41.7 ± 1.6	1.04 × 10^1^
Proposed				
*D* → ∞	0.059 ± 0.02	0.96 ± 0.01	44.8 ± 0.9	1.21 × 10^–2^
*D* = 2048	0.058 ± 0.02	0.96 ± 0.01	44.9 ± 0.9	1.20 × 10^–2^
*D* = 128	0.014 ± 0.00	**0.97** ± 0.01	45.3 ± 1.4	1.20 × 10^–2^
*D* = 64	0.015 ± 0.00	**0.97** ± 0.01	**45.4** ± 1.4	1.21 × 10^–2^

WGAN-VGG, CD, and PPFM are supervised methods. Noise2Void and proposed are unsupervised. BM3D is non-DL. ↓ means lower is better. ↑ means higher is better. Best results in bold

**Fig. 3:**
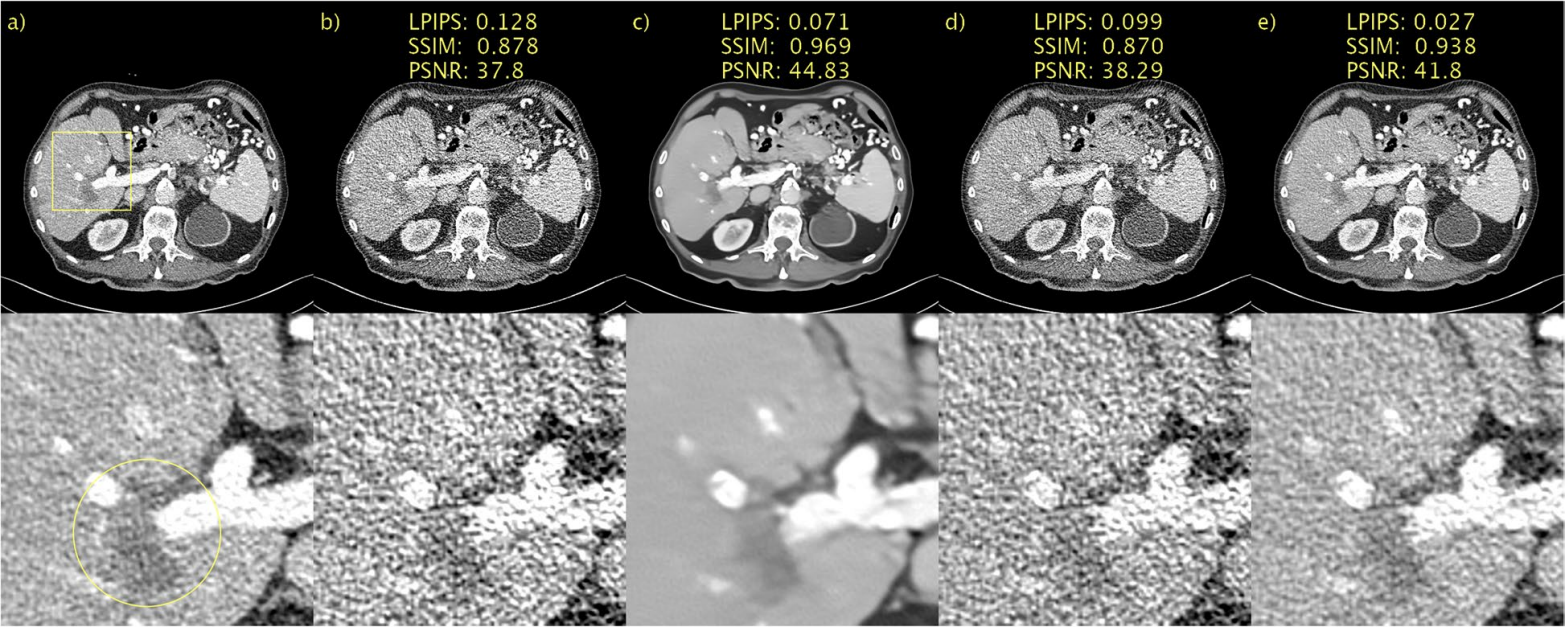
Ablation study of proposed sampler. **a** NDCT; **b** LDCT; **c** only hijacking; **d** only regularization; **e** hijacking and regularization. Yellow circle added to emphasize lesion. 1 mm-slices. Window setting [-160, 240] HU

The results of the ablation study of the proposed sampler are shown in Fig. 3. Recall the problem formulation, the objective is to obtain an estimate $\widehat{{\varvec{y}}}\in {\mathbb{R}}^{\rm N}$ of ${\varvec{y}}\in {\mathbb{R}}^{\rm N}$ based on a noisy observation $c=\mathcal{F}\left({\varvec{y}}\right)\in {\mathbb{R}}^{\rm N}$. By addressing this statistical inverse problem, the solution is a sample from the posterior $\widehat{{\varvec{y}}}\sim p\left({\varvec{y}}|{\varvec{c}}\right)$. Figures 3a and b show the NDCT (***y***) and LDCT (***c***) images, respectively, for ease of reference. In Fig. 3c, the regularization (τ = *T* − 1, *w* = 1) is hijacked but omitted. This results in an image that has been very aggressively denoised. This is a direct consequence of the small *T* and corresponding large step size. This represents a further clean demonstration of how SSIM and PSNR fail to adequately penalize blurring, as Fig. 3c appears blurry to a human observer, yet performs very well according to SSIM and PSNR. LPIPS, on the other hand, penalizes this heavily. In Fig. 3d, regularization occurs, but no hijacking (*τ* = 0, *w* = 0.5). In this case, an initial sample from a prior noise distribution was first used. The amount of regularization in this setting appeared to be excessive, and an image where $\widehat{{\varvec{y}} }\approx {\varvec{c}}$ was recovered. Finally, in Fig. 3e, hijacking and regularization (*τ* = *T* − 1, *w* = 0.5) were employed, resulting in a very pleasing image, where $\widehat{{\varvec{y}} }\approx {\varvec{y}}$. Consequently, there was a significant reduction (improvement) in LPIPS. Hijacking and regularization allows re-purposing of the pre-trained image generator for the task of image denoising. Conditioning on the LDCT (**c**) image via hijacking and regularization enforces a sample from the posterior $\widehat{{\varvec{y}}}\sim p\left({\varvec{y}}|{\varvec{c}}\right)$, where $\widehat{{\varvec{y}} }\approx {\varvec{y}}$ is the normal dose counterpart to ***c***. In other words, a Bayesian inference is performed.

**Fig. 4:**
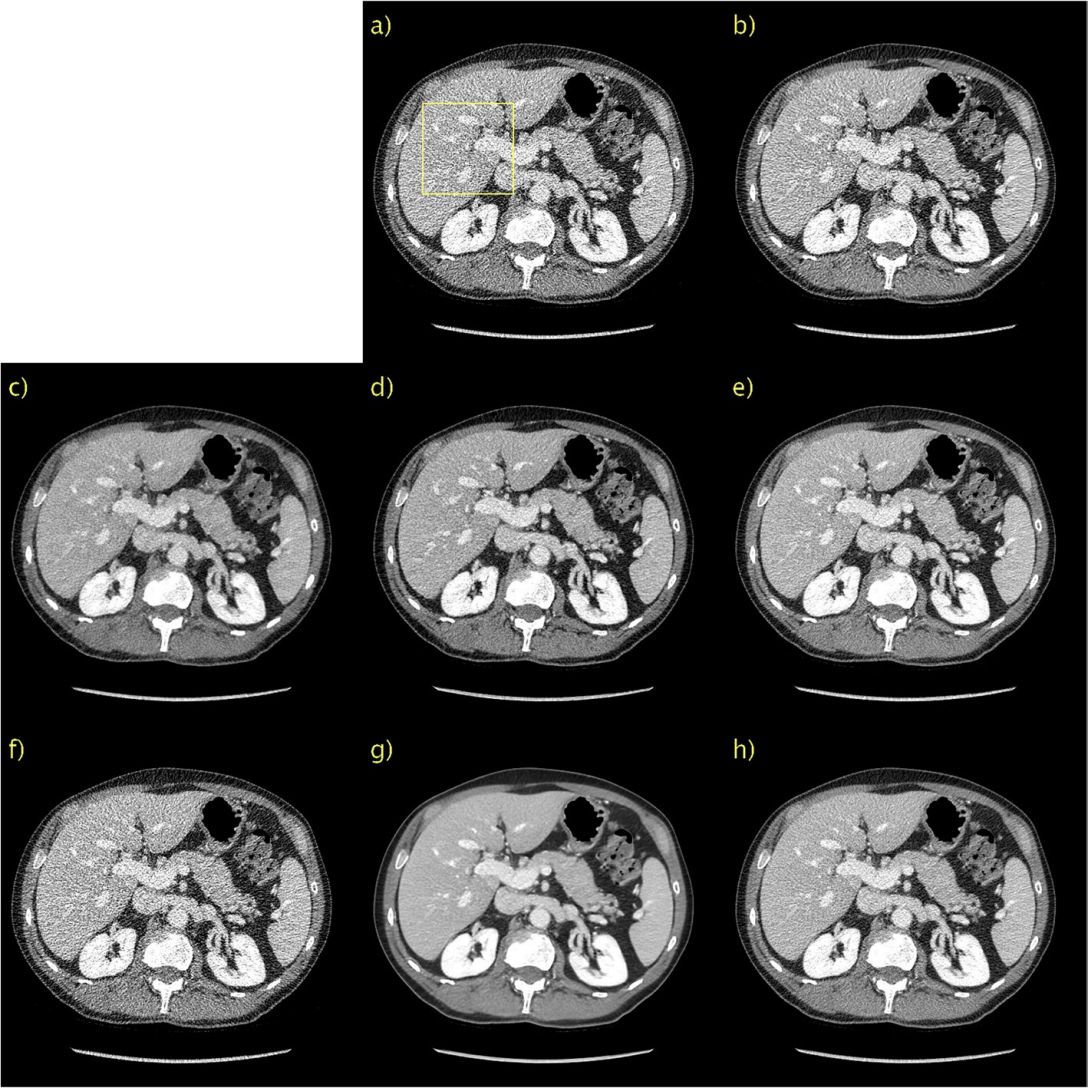
Results for first PCCT test case. **a** Unprocessed; **b** BM3D; **c** WGAN-VGG; **d** CD; **e** PPFM; **f** Noise2Void; **g** *D* → ∞; **h** *D* = 128. No ground truth available. Yellow box indicating ROI shown in Fig. 5. 0.42 mm-slices. Window setting [-160, 240] HU

**Fig. 5:**
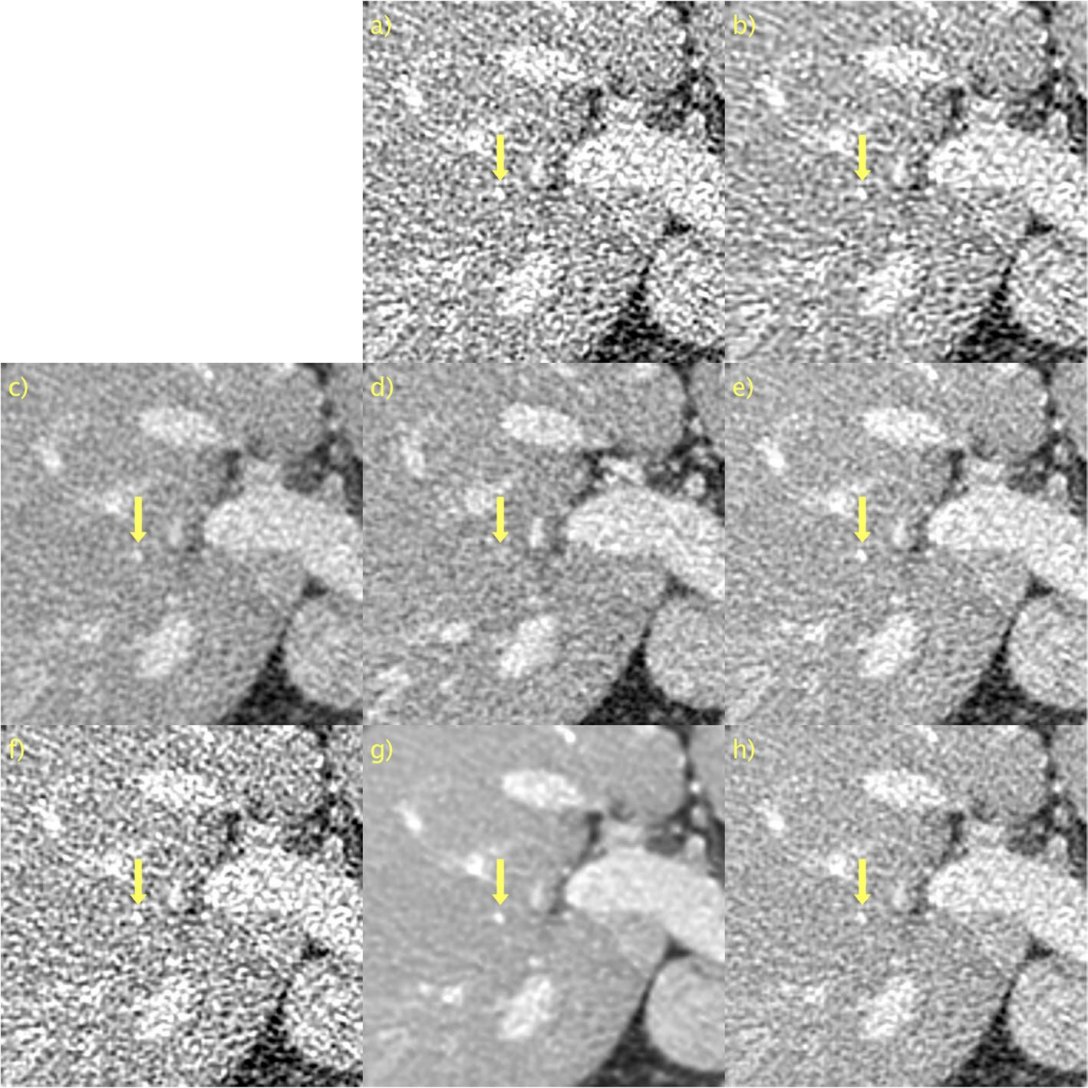
ROI in Fig. 4 magnified to emphasize details. **a** Unprocessed; **b** BM3D; **c** WGAN-VGG; **d** CD; **e** PPFM; **f** Noise2Void; **g** *D* → ∞; **h** *D* = 128. No ground truth available. Yellow arrow placed to emphasize detail. 0.42 mm-slices. Window setting [-160, 240] HU

Qualitative results for a representative slice in the first PCCT scan are shown in Fig. 4 with a magnification of the indicated ROI in Fig. 5. Owing to the lack of “ground truth” images, qualitative evaluation is used. It is also difficult to discriminate signals from noise in small, low-contrast details. Good performance is simply defined as accurately reproducing the unprocessed image, as shown in Figs. 4a and 5a, but with a lower noise level. BM3D, in Figs. 4b and 5b, appears to generalize poorly from LDCT data. This is likely due to the differences in noise characteristics; one would need to re-estimate *σ*_BM3D_. All other methods seem to generalize well in the sense that there are no major changes in performance. There is a significant performance gain for the proposed method with *D* = 128 compared to *D* → ∞. This was most visible in the magnified ROI in Fig. 7 as *D* → ∞, shown in Figs. 4g and 5g, is significantly more blurry than *D* = 128, shown in Figs. 4h and 5h. The yellow arrow indicating the detail of interest has been overlaid. Because there is no ground truth and a single slice is considered, it cannot be definitively stated that this is not just a noise spike. However, because it is clearly visible in an unprocessed image, it needs to be visible in the processed images. As can be seen, this is indeed the case with the notable exception of CD, shown in Figs. 4d and 5d. Moreover, the contrast of this detail appears to vary and seems to be much more well-defined for the proposed method with *D* = 128 than for WGAN-VGG, as shown in Figs. 4c and 5c. Hence, it appears that the proposed method, despite being unsupervised, can perform competitively, even when compared to supervised methods such as WGAN-VGG and CD.

**Fig. 6:**
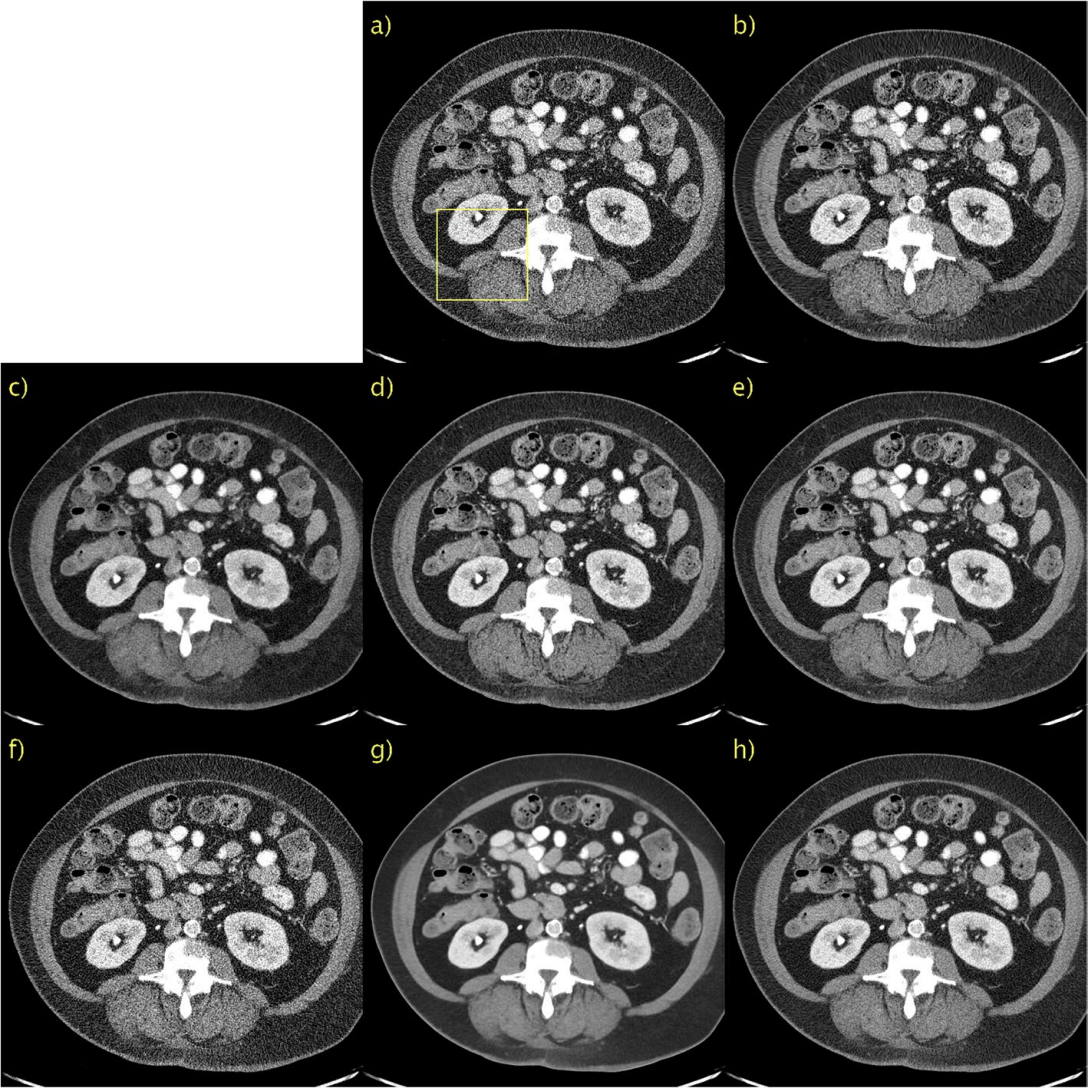
Results for second PCCT test case. **a** Unprocessed; **b** BM3D; **c** WGAN-VGG; **d** CD; **e** PPFM; **f** Noise2Void; **g** *D* → ∞; **h** *D* = 128. No ground truth available. Yellow box indicating ROI shown in Fig. 7. 0.42 mm-slices. Window setting [-160, 240] HU

**Fig. 7:**
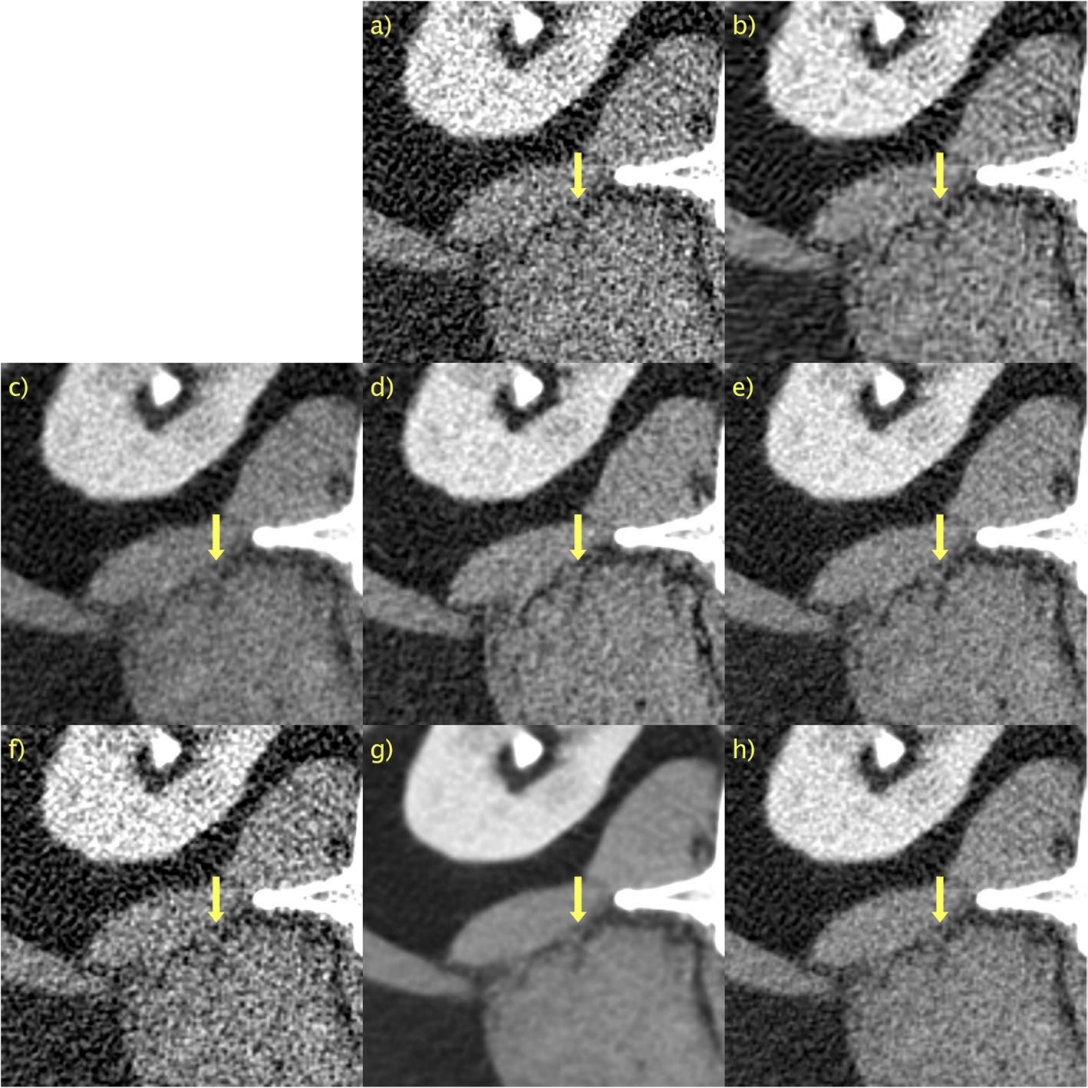
ROI in Fig. 6 magnified to emphasize details. **a** Unprocessed; **b** BM3D; **c** WGAN-VGG; **d** CD; **e** PPFM; **f** Noise2Void; **g** *D* → ∞; **h** *D* = 128. No ground truth available. Yellow arrows placed to emphasize detail. 0.42 mm-slices. Window setting [-160, 240] HU

The results of a representative slice in the second PCCT scan are shown in Figs. 6 and 7. Again, no “ground truth” is available. BM3D, in Figs. 6b and 7b, appeared to perform better than the first PCCT test case. The denoising performance is now more aligned with that observed for the LDCT validation data. This difference in performance is likely due to the differences in noise characteristics and a lack of generalization. The lack of generalization does not appear to be an issue for any other method, as the performance is consistent across the validation and test data. A yellow arrow is again placed to indicate the details of interest, in this case, the fat in the back muscle. The contrast is difficult to assess qualitatively when comparing the proposed method with *D* → ∞, shown in Figs. 6g and 7g, and *D* = 128, shown in Figs. 6h and 7h, owing to the large difference in the noise level. *D* → ∞ is definitely oversmoothed, and thus blurry; however, the contrast of this particular detail seems to be fairly well preserved. The proposed method with *D* = 128 again performs very competitively compared with WGAN-VGG, as shown in Figs. 6c and 7c, as can be seen when considering the contrast of fat and muscle.

The results from the visual assessment are summarized in Table 3. Statistical significance was evaluated using a single-sample, two-tailed t-test without applying any multiple-comparison-based correction. From the total score in the table, it can be observed that the proposed method with *D* → ∞ performs on par with CD, whereas PPFM and *D* → ∞ are assigned somewhat lower scores and Noise2Void, BM3D, and WGAN-VGG exhibit the worst performances. The fact that *D* → ∞ performs on par with CD is remarkable because the latter is a supervised method, and therefore has access to more data during training. Furthermore, the proposed method has a smaller number of parameters than CD, which is reflected in its shorter inference time (Table 2). It can also be noted that the value *D* = 128 gives the optimal LPIPS, which measures the image fidelity in the feature space, whereas the visual assessment gives a higher score to the *D* → ∞ method because radiologists prefer its stronger noise suppression. The fact that adjusting *D* allows the optimization of the method for different performance metrics underscores the flexibility of the proposed method.

**Table 3:** Average and standard deviation of the visual assessment score of the different denoising methods relative to CD [37], over the two observers and the three cases

Method	Noise	Contrast	Sharpness	Artifact	Overall image quality	Total score
BM3D [42]	-1.50 ± 0.55**	-0.67 ± 0.52*	-1.17 ± 0.41***	-1.00 ± 0.63*	-1.50 ± 0.55**	-1.17 ± 0.59***
WGAN-VGG [9]	-0.50 ± 0.84 ns	-1.00 ± 0.00***	-1.33 ± 0.52**	-0.50 ± 0.55 ns	-1.17 ± 0.41***	-0.90 ± 0.61***
PPFM [19]	0.00 ± 0.63 ns	-0.50 ± 0.55 ns	-0.17 ± 0.98 ns	-0.50 ± 0.55 ns	-0.17 ± 0.75 ns	-0.27 ± 0.69*
Noise2Void [44]	-1.67 ± 0.52***	-1.00 ± 0.63*	-1.33 ± 0.52**	-0.50 ± 0.55 ns	-1.67 ± 0.52***	-1.23 ± 0.68***
Proposed						
*D* → ∞	1.50 ± 0.55**	-0.33 ± 0.52 ns	-0.67 ± 0.82 ns	-0.17 ± 0.75 ns	-0.33 ± 1.21 ns	0.00 ± 1.08 ns
*D* = 128	-0.17 ± 0.75 ns	-0.83 ± 0.41**	-1.00 ± 0.00***	-0.33 ± 0.52 ns	-1.00 ± 0.00***	-0.67 ± 0.55***

For the ‘noise’ and ‘artifact’ categories, lower noise and less artifacts gives higher score. The “total score” column is the average score over the other five categories and the standard deviation over all assessments. *, ** and *** denotes significance at the *p* = 0.05, 0.01 and 0.001 level. “ns”: not significant

## Discussion

Because image denoising was of interest, the simplest possible data consistency (or regularization) step was selected: the identity map. An interesting direction for future research is to determine how well the proposed method generalizes to other inverse problems. It can be surmised that updating only the regularization step is sufficient, as is the case with similar techniques based on diffusion models [34, 35]. One interesting application is the combination of image denoising and super-resolution. However, it is possible that one also needs to update the hijacking method, because injecting the condition image directly into the sampling process may fail to generalize beyond the problem of image denoising. Even within the two tasks of denoising LDCT and PCCT images, noise characteristics vary widely. CT images are routinely reconstructed using different kernels, slice thicknesses, fields of view, and matrix sizes. These factors may have resulted in reconstructed images with significantly different noise characteristics. As shown in ref. [47], this may adversely affect the performance of image denoising techniques. Hence, it is possible that one needs to update the hyperparameters in the sampling algorithm, including the consistency step, to attain good performance over a wide range of settings.

Another interesting avenue for future research is the extension of the proposed method to 3D denoising. Given the structure of CT data, 2D denoising discards an abundance of rich information by not considering adjacent slices. It is possible that this additional information can aid in the recovery of more details observed in NDCT images from LDCT data. In particular, using information from adjacent slices may help better differentiate noise from signals. Extending the proposed method to 3D denoising has two effects. First, the network can be retrained using 3D data. The benefit of this approach is that it allows the network to optimize the use of information from adjacent slices. However, a disadvantage of this approach is that it requires retraining. Another possibility is to update only the data consistency step. One can keep the learned prior retained from the 2D data, thus not requiring any retraining, and combined with a regularization step that utilizes information from adjacent slices. Finally, the two approaches were combined. In future studies, the proposed method will be extended for 3D denoising.

In the current generation of PCCT scanners, spectral (materially resolved) CT are usually of interest. Hence, extending this method to spectral cases is of interest. Similar to the 3D case, one could allow the network to accept more channels, where the channels could be material-basis images or virtual monoenergetic images at two or more energy levels. Another possibility is to train two separate networks for two different material bases or virtual monoenergetic images at different energy levels.

Although strong correlation between LPIPS [41] and visual image quality can be observed, it should be noted that LPIPS is computed using a network trained on RGB natural images. Relative to natural images, grayscale CT images should be considered outside the distribution. Nevertheless, the results indicate that using these selection criteria yields visually pleasing images in a LDCT dataset. Furthermore, the results for the PCCT data indicate that the network with these sampling hyperparameters generalized well. These empirical findings strongly suggest the intrinsic nature of LPIPS features. One possible way to make LPIPS more interpretable is to retrain the underlying network in LPIPS on a large dataset of CT/PCCT images. This important work is beyond the scope of this study and is left to future research.

## Conclusions

In practice, paired data are typically unavailable for denoising CT images. In this study, an unsupervised version of the PPFM [19] was proposed and it was demonstrated that despite imposing a significantly laxer data requirement, there is only a small drop in the overall performance. To achieve this, a PFGM++ [29] trained in an unsupervised manner for unconditional generation with a sampling scheme that enforces consistency was combined with the input or condition image to enable sampling from the desired posterior. The proposed method includes a corresponding method based on diffusion models (EDM [27]), and it was demonstrated that PFGM++, with *D* as an additional hyperparameter, yields significant performance gains. The results indicate competitive performance compared with popular supervised, unsupervised, and non-DL-based image denoising techniques, including state-of-the-art diffusion-style models with NFE = 1, consistency models, clinical LDCT data, and clinical images from a prototype PCCT system.

## Data Availability

Code used for this project is available at: https://github.com/dennishein/pfgmpp_PCCT_denoising. Mayo low-dose CT data used are available at: https://aapm.app.box.com/s/eaw4jddb53keg1bptavvvd1sf4x3pe9h/folder/144226105715.

## Abbreviations

CT: Computed tomography
PCCT: Photon-counting CT
DL: Deep learning
NFE: Number of function evaluations
ODE: Ordinary differential equation
CD: Consistency distillation
PFGM: Poisson flow generative models
AAPM: American Association of Physicists in Medicine
NDCT: Normal-dose CT
LDCT: Low-dose CT
LPIPS: Learned Perceptual Image Patch Similarity
ROI: Region-of-interest
SSIM: Structural similarity index
PSNR: Peak signal-to-noise ratio
CNN: Convolutional neural network
